# A Pivotal Role of Nrf2 in Neurodegenerative Disorders: A New Way for Therapeutic Strategies

**DOI:** 10.3390/ph15060692

**Published:** 2022-05-31

**Authors:** Sibel Suzen, Paolo Tucci, Elisabetta Profumo, Brigitta Buttari, Luciano Saso

**Affiliations:** 1 Department of Pharmaceutical Chemistry, Faculty of Pharmacy, Ankara University, Tandogan, 06100 Ankara, Turkey; 2 Department of Clinical and Experimental Medicine, University of Foggia, Via Napoli, 20, 71122 Foggia, Italy; paolo.tucci@unifg.it; 3 Department of Cardiovascular and Endocrine-Metabolic Diseases and Aging, Istituto Superiore di Sanità, Viale Regina Elena 299, 00161 Rome, Italy; elisabetta.profumo@iss.it (E.P.); brigitta.buttari@iss.it (B.B.); 4 Department of Physiology and Pharmacology ‘‘Vittorio Erspamer”, Sapienza University of Rome, P. le Aldo Moro 5, 00185 Rome, Italy; luciano.saso@uniroma1.it

**Keywords:** Alzheimer’s disease, Huntington’s disease, Parkinson’s disease, ALS, Nrf2, oxidative stress, antioxidant, neurodegenerative

## Abstract

Clinical and preclinical research indicates that neurodegenerative diseases are characterized by excess levels of oxidative stress (OS) biomarkers and by lower levels of antioxidant protection in the brain and peripheral tissues. Dysregulations in the oxidant/antioxidant balance are known to be a major factor in the pathogenesis of neurodegenerative diseases and involve mitochondrial dysfunction, protein misfolding, and neuroinflammation, all events that lead to the proteostatic collapse of neuronal cells and their loss. Nuclear factor-E2-related factor 2 (Nrf2) is a short-lived protein that works as a transcription factor and is related to the expression of many cytoprotective genes involved in xenobiotic metabolism and antioxidant responses. A major emerging function of Nrf2 from studies over the past decade is its role in resistance to OS. Nrf2 is a key regulator of OS defense and research supports a protective and defending role of Nrf2 against neurodegenerative conditions. This review describes the influence of Nrf2 on OS and in what way Nrf2 regulates antioxidant defense for neurodegenerative conditions. Furthermore, we evaluate recent research and evidence for a beneficial and potential role of specific Nrf2 activator compounds as therapeutic agents.

## 1. Introduction

Oxidative stress (OS) is a physiopathological state characterized by an imbalance between reactive oxygen (ROS) and nitrogen species (RNS) generation and cellular antioxidant capacity. Excess ROS formation causes critically important changes in cellular biomolecules, such as proteins, DNA, and lipids. There are numerous studies that confirm a major relationship between OS and neurodegenerative disorders [1,2] like Alzheimer’s disease (AD) [3], Huntington’s disease (HD) [4], Parkinson’s disease (PD) [5], Multiple sclerosis (MS) [6] and Amyotrophic Lateral Sclerosis (ALS) [7].

ROS/RNS are generated from many different sources in multiple compartments within the cell, either physiologically or because of exposure to toxic or pathologic conditions [8]. One of the most active types of ROS, superoxide (O_2_^−^), is produced by the one-electron reduction of O_2_ in mitochondria. Superoxide can also be produced by a family of NADPH oxidases (NOXs), using oxygen and NADPH as substrates in which superoxide is promptly disposed of [9]. Another important side-product of mitochondrial oxidative phosphorylation is hydroxyl radical (**^.^**OH). This is very unsteady, extremely reactive, and produces several reactive aldehydes from membrane lipid peroxidation (LP) that eventually cause cell death. Moreover, the ROS hydrogen peroxide (H_2_O_2_), is rapidly formed in the cytoplasm by superoxide dismutase 1 (SOD1), while the H_2_O_2_ outside the cell is generated by extracellular superoxide dismutase 3 (SOD3). To end up, H_2_O_2_ can be produced as a by-product during *β*-oxidation of fatty acids by cytochrome P450s. Although H_2_O_2_ is relatively more stable and less reactive, in the presence of Fe^2+^ or Cu^+^ (Fenton reaction), it can be transformed into hydroxyl radical [10].

The main RNS is ONOO^−^ that rapidly decays into HO•, nitrogen dioxide radical (NO_2_•), and nitryl cation (NO_2_^+^) [11]. All of these are neurotoxic.

There are multiple steps in the production of the OS and in the imbalance of the endogenous cellular defense in neuronal cells that could be targeted therapeutically in the processing of neurodegenerative diseases.

Nuclear factor (erythroid-derived 2)-like2 (Nrf2), is a transcriptional factor associated with the essential defense mechanism of the cells against OS inducing expression of cytoprotective genes [12,13]. Moreover, the Nrf2 is crucial for blood cell differentiation and for the induction of a set of drug-metabolizing enzymes [14]. Proteins upregulated by Nrf2 signaling include heme oxygenase-1 (HO-1), SOD1, catalase, and enzymes involved in glutathione (GSH) metabolism, such as glutathione S-transferase (GST), glutathione cysteine ligase modifier subunit, and glutathione cysteine ligase catalytic subunit (GCLC) [15,16].

The capacity of Nrf2 to control intermediary metabolism and mitochondrial action leads that Nrf2 activation is a smart and comprehensive approach to the management of neurodegenerative disorders [17]. This review discusses the importance of oxidative stress in neurodegenerative diseases and the advantages associated with targeting the Nrf2 pathway as a transcriptional antioxidant and cytoprotective response and highlights new candidate therapeutics that have been developed to able to recover oxidative damage and neuroinflammation through the Nrf2 signaling pathway activation.

## 2. Oxidative Stress and Neurodegenerative Conditions

The brain is very predisposed to OS because of low antioxidant levels, such as catalase and GSH, but also because neurons and microglia produce a large amount of OS [18,19]. Moreover, the brain is rich in unsaturated lipids, Fe^2+^ or Cu^+,^ an ideal environment for LP and ferroptosis [20,21].

Ferroptosis is described as non-apoptotic, iron-dependent, oxidative cell loss. It plays a significant part in the brain and neurological disorders [22]. Ferroptosis is reliant on excess iron accumulation, which is a critical factor of LP [23]. Neurodegenerative disorders cell death processes, which are closely related to excess buildup of iron and LP in the brain [24]. This excess iron accumulation in neurodegenerative disorders caused OS generation, mitochondrial activity failure, over the formation of ROS as well as damage to DNA [25]. It is stated that ferroptosis is organized by NRF2 and BACH1. NRF2 and BACH1 work by stimulating or preventing the expression of genes in the pathways of ferroptosis [26].

Ferroptosis is considered a significant preclinical mark of AD. LP and excess iron worsen amyloid β peptide and tau aggregation, which are significant in AD pathogenesis [27]. Recent research showed that BACH1/NRF2 proportion in the modulation of the antioxidant defense, a valued approach therapeutically to investigate molecules could utilize neuroprotective activity. For example, Down syndrome (DS) is a multifaceted genetic disease described by BACH1 gene triplication that possible consequences in the damage of NRF2 causing augmented OS. Pagnotta et al. [28] suggested that overproduction of BACH1 modifies the BACH1/NRF2 proportion and interrupts the initiation of antioxidant response genes eventually causing excessive oxidative damage. They found that the theory that BACH1 triplication in DS is involved in the modification of redox homeostasis.

Research has recognized the part of ferroptosis in neurotoxicity and brain injuries, proposing the pharmacological prospective of ferroptosis inhibition [29]. Diseases and some other biological processes associated with ferroptosis neurodegeneration [30,31], autoimmune diseases [32], and a rare genetic neurological disorder called Pelizaeus-Merzbacher Disease [33]. Ferroptosis is a form of regulated necrosis that induces an increase in lethal levels of phospholipid hydroperoxides from polyunsaturated fatty acid (PUFA) [34]. This process is iron-dependent and causes membrane perforation and other damages within cellular membranes [35,36].

A further source of OS is the neurotransmitters with catechol groups like dopamine that generate hydrogen peroxide when is metabolized by monoamine oxidases (MAO) [37].

The areas of the brain more susceptible to OS are the cerebral cortex, the hippocampus, and the striatum [20,38]. In the neurodegenerative conditions, aggregates of misfolded proteins and mitochondrial dysfunction are the main prompter of ROS release.

The role of OS in general and of ROS, in particular, has been recognized using many different animal models and a large variety of cultured cells. Moreover, the OS increase is a factor age-dependent that promotes pathological alterations that can trigger neurodegenerative diseases [39]. An antioxidant defense system to fight the effects of OS in the brain is the Nrf2, the chief regulator of redox homeostasis by triggering the antioxidant enzyme system and regulating both mitochondrial function and biogenesis [40,41]. These activities have made the Nrf2 a promising therapeutic target investigated in pre-clinical and clinical studies [42]. Furthermore, Nrf2 has also been found to show anti-inflammatory activity adding importance to its role in neurodegenerative diseases [43]. In fact, neuroinflammation is described as the inflammatory response of the central nervous system (CNS) against harmful stimuli closely related to many neurodegenerative conditions [44]. A number of studies have shown that the release of pro-inflammatory cytokines is due to inflammasome, a multiprotein complex triggered by receptors as the nucleotide-binding domain and leucine-rich repeat-containing receptors (NLRs) family [45]. Nevertheless, in humans, the mechanism of the inflammatory response is not entirely clarified. Redox balance is affected by the occurrence of neuroinflammation or unbalanced mitochondrial activity [46].

## 3. The Nrf2-ARE Pathway as a Therapeutic Target

Nrf2 organizes cellular protection mechanisms against oxidants via modifying the expression of more than 500 genes that are related to antioxidants, detoxification pathways, or metabolic enzymes. Kelch-like ECH-associated protein (KEAP1) is one of the main regulators of Nrf2 protein stability. Under normal homeostatic conditions, Nrf2 is located in the cytosol and binds KEAP1 [2].

Nrf2 is one of the members of the cap “n” collar (CNC) subfamily of basic-region leucine zipper (bZIP) transcription factors along with Nrf1, Nrf3, NF-E2 p45 subunits and the less related factors BTB domain and CNC homolog 1 and 2 (Bach1 and Bach2) [47,48].

Molecular structure characterization of Nrf2 revealed seven functional domains, called Nrf2ECH homology (Neh) domains 1–7 with distinct functions [49]. Neh1 domain is responsible for the binding to DNA [50] and contains a nuclear localization signal (NLS) for Nrf2 translocation from the cytoplasm to the nucleus [51]. The Neh2 domain is involved in the interaction with KEAP1, the main Nrf2 repressor with an essential part in regulating the Nrf2 signaling pathway [52]. Neh3 is responsible for the activation of the antioxidant response element (ARE), a cis-regulatory element that primarily responds to oxidative stress inducers. Neh4 and Neh5 are involved in the binding with different “cAMP (cyclic Adenosine MonoPhosphate) response element-binding” (CREB) proteins and activate transcription [53,54]. Neh6 domain is a negative regulatory domain that promotes Nrf2 ubiquitination [55]. The Neh7 domain inhibits the Nrf2-ARE signaling pathway by promoting the binding of Nrf2 to the Retinoic X Receptor (RXR) and disrupting binding between CBP (CREB-binding protein) and the Neh4 and Neh5 domains [56].

In physiological conditions, Nrf2 is retained in the cytoplasm by the KEAP1/Cullin-3/E3 Ubiquitin-Protein Ligase RBX complex, and undergoes proteasomal degradation, thus maintaining the expression of ARE-responsive genes at basal levels [57]. When cells are exposed to pro-oxidant conditions, they activate the Nrf2/KEAP1/ARE pathway [58,59]. Three cysteine residues (Cys151, Cys273, and Cys288) of KEAP1 are important for Nrf2 degradation. When these residues are oxidized, Nrf2 releases [60]. During OS, ROS causes structural changes on KEAP1, inhibiting its binding to Nrf2. Furthermore, the p62 protein, whose expression is prompted by ROS, also helps the stimulation of Nrf2 by docking straight onto KEAP1 through a KEAP1 binding area. This action results in blocking the binding between KEAP1 and Nrf2 [61,62].

Oxidative or electrophilic challenges distract the complex between Nrf2 and Keap1, leading to the translocation of Nrf2 to the nucleus. This allows Nrf2 to heterodimerise with musculoaponeurotic fibrosarcoma proteins (MAFs) and to attach to ARE in the promoter area of target genes [63,64]. Nrf2 is constantly produced and degraded, showing a half-life of just 20–30 min. The heterodimer identifies ARE that is existing in the regulatory areas of around 250 ARE-genes [65]. The sequence ARE was first identified on the promoter of the rat gene encoding the GST A2 subunit (GST A2) [66]. Nrf2 binds to the cis-acting enhancer ARE sequence (core sequence: 5′-TGACNNNGC-3′) existing in promoters of genes [67]. Of note, the *nrf2* gene includes two ARE-like sequences in its promoter so that Nrf2 is able to autoregulate itself and make ARE-mediated gene expression longer [68].

The ARE is situated in the promoter area of a number of genes encoding phase II detoxifying enzymes, antioxidant enzymes, and proteins such as NAD(P)H:quinone oxidoreductase 1 (NQO1), GST, glutamate-cysteine ligase (GCL), HO-1, thioredoxin reductase-1, and thioredoxin [69].

Since the ARE core sequence has similarities to the sequence regulated by activator protein 1 (AP-1), it is possible that members of the Jun and Fos families of transcriptional factors could have a role in the transcriptional activation of the rat GST A2 subunit (*GST A2*) and quinone reductase (*QR*) genes. This suggestion was supported by the studies that Jun and Fos family members can be activated by OS [70,71]. Hence, Nrf2 is a transcription factor that responds to OS by binding to ARE in the promoter of genes coding for antioxidant enzymes and proteins for GSH synthesis [72,73].

NADPH is an essential cofactor for numerous drug-metabolizing enzymes and antioxidant systems, such as cytochromes p450 (CYP) enzymes and the Nrf2 target NQO1 [74]. Nrf2 supports NADPH production through the positive regulation of the principal NADPH-generating enzymes.

**Figure 1:**
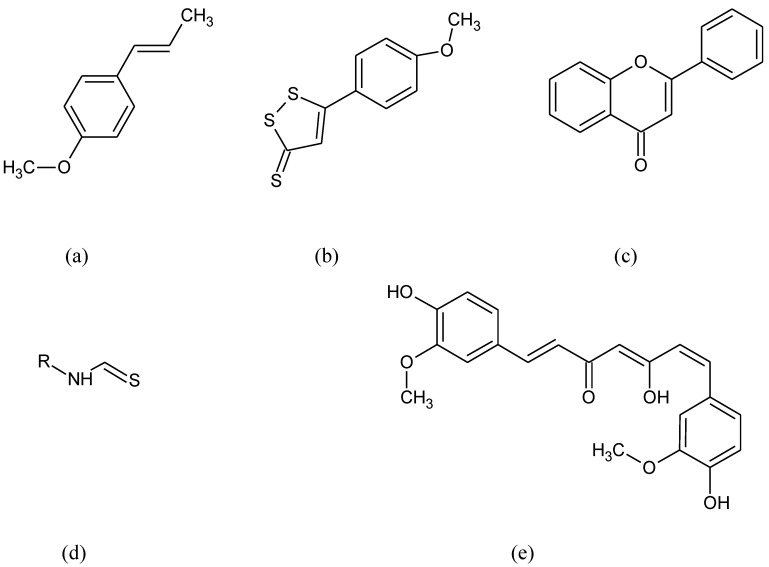
Chemical formula of (**a**) anetol, (**b**) anethole trithione, (**c**) flavon, (**d**) isothiocyanates, (**e**) curcumin.

Many different compounds like anethole derivatives such as anethole trithione, dithiolethiones, curcumin, isothiocyanates, caffeic acid, phenethyl esters, flavon derivatives, and triterpenoids (Figure 1) have been found to stimulate ARE and the phase II detoxifying enzymes [75,76].

Recently, a body of literature has demonstrated that numerous food compounds protect against ferroptosis via activating Nrf2 [34].

The capability of the Nrf2 pathway to regulate genes related to antioxidant protection, autophagy, and proteasome activation has been drawing attention to the importance of Nrf2 activator compounds as therapeutic approaches for neurodegenerative diseases [77].

## 4. Neurodegenerative Diseases Related to Oxidative Stress and Nrf2 Activation

### 4.1. Nrf2 in Alzheimer’s Disease

AD is an age-related neurodegenerative disease mainly defined by amnesia, diminished executive functions, and behavioral alterations that are characterized by two molecular hallmarks: plaques of beta-amyloid (Aβ) and tau protein. The latter one is phosphorylated, and this process produces neurofibrillary tangles increasing aggregation and toxicity of the protein in the cell bodies of neurons. Some tau proteins are linked to amyloid plaques producing neuritic plaque [78].

**Figure 2:**
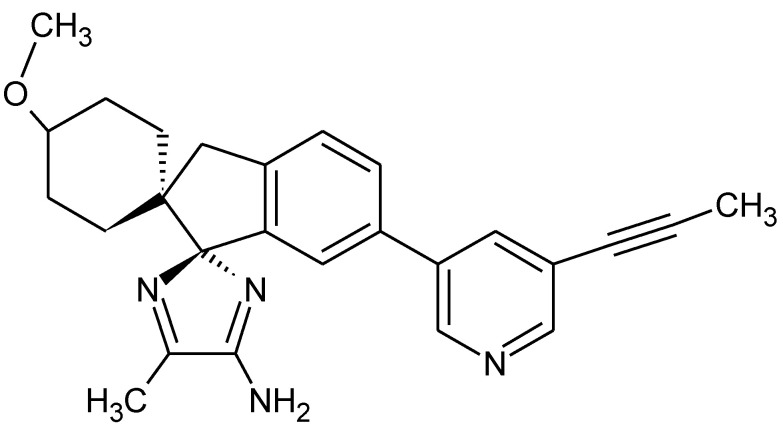
Chemical formula of lanabecestat.

Finding a drug molecule for the treatment of AD is still ongoing, as the pathogenesis of AD is not entirely described. The drugs lanabecestat, (an inhibitor of beta-secretase 1 cleaving enzyme (BACE1), a fundamental key for the generation of amyloid-β peptides in the neurons) (Figure 2), crenezumab (a monoclonal antibody against multiple forms of aggregated Aβ), and solanezumab (a humanized monoclonal IgG1 antibody directed against the mid-domain of the Aβ peptide that recognizes its soluble monomeric state) have failed to show expected efficacy in the clinical trials. In 2021, the U.S. Food and Drug Administration approved aducanumab, a new monoclonal antibody against a conformational epitope found on Aβ. Unfortunately, it is unclear how effective it is in improving patients’ cognitive skills. The European Medicines Agency rejected a marketing application on 16 December 2021.

It is worthwhile to study alternative therapeutic strategies to drugs based on the inhibition of protein aggregation. The researchers have identified some areas that have received relatively little attention yet but may hold the seeds of new hope for an efficacy therapy. One of these areas is the brain’s OS reactions [79]. Exposure to excess OS affects tau hyperphosphorylation by promoting the activity of p38 MAPK [80].

Histopathological research showed that in AD brain patients, mitochondria and NOX are the two major sources generating ROS [81]. Tau protein also leads to escalating ROS formation which was the main result of energy dysfunction of mitochondria. Abnormally phosphorylated tau protein dissociates from microtubules and aggregates into neurofibrillary tangles thus altering mitochondrial function and promoting ROS burst [82]. Therefore, ROS produces damage to mitochondrial DNA (mtDNA) [83].

Besides mitochondria, one of the significant findings is neuroinflammation due to extensive activation of astrocytes and microglial cells detected in tau-related conditions. Glial activation is associated with the high levels of interleukin-1β (IL-1β) and cyclooxygenase-2 (COX2). It proves the involvement of glial activation in AD and suggests a link between activated glial cells and AD pathogenesis [84]. Hence, preventing the formation of neuroinflammation has been described to be a promising approach in AD treatment.

The level of Nrf2 is observed to be reduced in AD patients [85] and a significant negative correlation between Nrf2 deficits and AD has also been reported [86,87]. Research showed that Nrf2 activation not only applies anti-oxidative properties but also reduces neuroinflammation [88]. Nrf2, directly and indirectly, influences changes in autophagy in vivo and in vitro [89,90]. Moreover, activation of Nrf2 by genetic and pharmaceutical interventions leads to a neuroprotective role in AD patients [91]. Thus, Nrf2 signaling is a significant regulator of neuroinflammation in AD, which provide an awareness of the potential of Nrf2 regarding finding other beneficial therapeutical strategies for AD [92].

**Figure 3:**
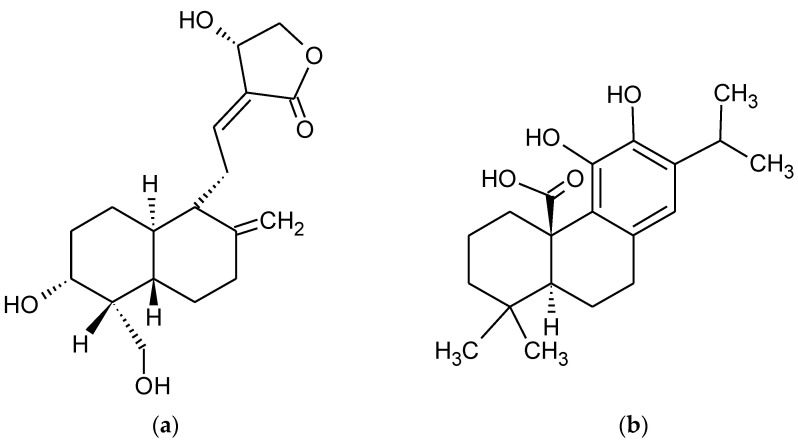
Chemical formula of (**a**) Andrographolide (Andro) and (**b**) carnosic acid (CA).

It has been shown that andrographolide (Andro) (Figure 3a) may bind to a spectrum of protein targets including “nuclear factor kappa-light-chain-enhancer of activated B cells” (NF-_K_B) and actin by covalent modification [93]. Andro is the principal bioactive chemical constituent of *Andrographis paniculata* (Acanthaceae), which exhibits a wide range of biological activities including anti-inflammatory, antioxidant, and promising antidiabetic potential [94,95]. The Andro significantly ameliorated cell death due to Aβ1–42 insult through the activation of autophagy and the Nrf2-mediated p62 signaling pathway in PC12 cells. In the early stage of AD, autophagy promotes the clearance of Aβ and Tau. With the disease progression, Aβ and Tau are continuously produced and accumulated, thus determining autophagy dysfunction. Nrf2 promotes the expression of the regulated autophagy marker SQSTM1/P62 and of NDP52, the receptor that promotes selective autophagy by the interaction with LC3 and cargo on autophagosome, thus supporting the clearance of tau [96]. These mechanisms could be useful also for other neurodegenerative diseases (see below, Parkinson’s disease paragraph).

In a transgenic mouse model (J20 Tg) with mild AD phenotype expression (high levels of amyloid aggregates), presymptomatic administration of Andro prevented the reduction of cellular energy metabolism markers, improved cognitive performance, restored the deficiencies at the synaptic level, and restored the length of synapses. Of note, Andro is a canonical Wnt signaling activator. Wnt signaling is a pathway involved in several processes during the development and maintenance of the adult central nervous system. The loss of Wnt signaling function has been associated with neuronal dysfunction in AD. The data obtained in the J20 Tg mouse model support the idea that the Andro activation of Wnt signaling during presymptomatic stages could represent an interesting pharmacological strategy to delay the onset of AD [97].

The further results indicated that this neuroprotective effect may be mainly due to the inhibition of NO, TNF-α, IL-6, ROS, and iNOS production, and to the enhanced expression of the anti-inflammatory marker CD20, determined by the suppression of nuclear translocation of NF-κB as well as the activation of Nrf2 and HO-1 [98].

A study carried out in the aged Chilean rodent *Octodon degus* that have been proposed as a potential “natural” model for sporadic AD, demonstrated that intraperitoneal treatment with Andro significantly reduced Aβ burden, astrogliosis, and interleukin-6 levels in brains. Furthermore, it reduced 4-hydroxynonenal and N-tyrosine adducts levels, thus demonstrating that Andro is able to induce a relevant reduction of oxidative stress [99]. In a recent study, ref. [100] evaluated Andro neuroprotective activity and its potential for effects on AD using the aluminum maltolate (Al(mal)3)-induced neurotoxicity in PC12 cells. Andro significantly increased the viability of Al(mal)3-treated cells. Moreover, it decreased the expression of APP, BACE1, and Keap1 proteins while increasing the protein and mRNA expression of Nrf2. Silencing p62 or Nrf2 can significantly reduce the protein and mRNA expression of Nrf2 and p62 under co-treatment with Andro and Al(mal)3. These results suggest that Andro could be a promising therapeutic tool to contrast neurotoxicity by regulating the p62-mediated Keap1/Nrf2 pathway [101].

It was proved that electrophilic molecules stimulate the Keap1/Nrf2 pathway when they interact with thiol groups on Keap1, thus causing the Nrf2 accumulation in the cytoplasm and it enters into the nucleus, where it binds to the ARE on the promoters of phase 2 genes [102]. The Carnosic Acid (CA) (Figure 3b) is a natural product found as a pro-electrophilic molecule that is changed to its active form by OS. This form triggers the Keap1/Nrf2 transcriptional pathway to create phase 2 antioxidant enzymes [103] found that histologically, CA increased dendritic and synaptic markers, and decreased astrogliosis, A*β* plaque number, and phospho-tau staining in the hippocampus, thus explaining CA therapeutic benefits in rodent AD models.

**Figure 4:**
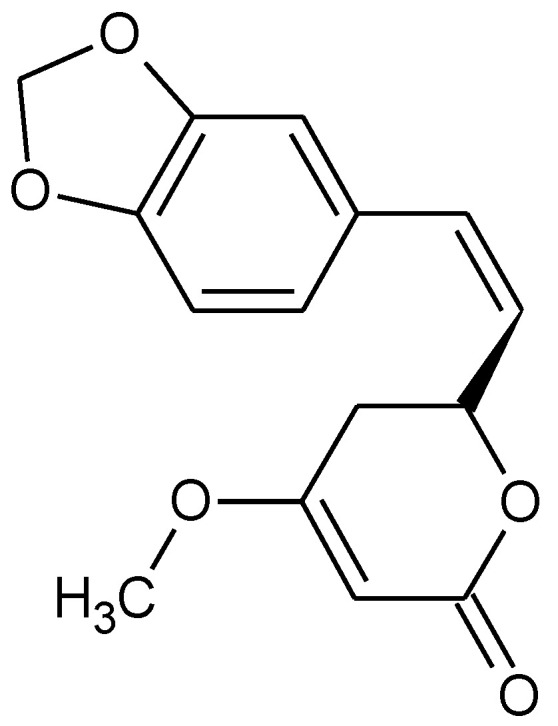
Chemical formula of methysticin.

Methysticin is a kavalactone derivative found in the kava plant that belongs to the piperaceae family. The administration of this compound for 6 months to transgenic APP/Psen1 mice (a mouse model of AD), determined the Nrf2 pathway activation in the hippocampus and cortex. Furthermore, it significantly reduced microgliosis, astrogliosis, oxidative damage, and the secretion of the pro-inflammatory cytokines TNF-α and IL-17 [104] (Figure 4).

Sulfur is widely existent in natural products and synthetic organic compounds such as organosulfur, which are often associated with a multitude of biological activities since they have innate antioxidant potential, and some are currently being evaluated in clinical trials. O-benzothiazole, in which the benzene ring is fused to the 4,5-positions of the thiazolerganosulfur compounds continues to garner increasing amounts of attention in the field of medicinal chemistry, especially in the development of therapeutic agents for AD [105].

**Figure 5:**
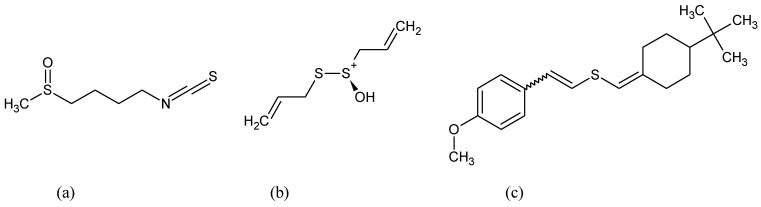
Chemical formula of (**a**) sulforaphane, (**b**) allicin and (**c**) [(4-*tert*-butylcyclohexylidene)methyl] (4-methoxystyryl)sulfide (BMMS).

Sulforaphane (Figure 5a) is a compound within the isothiocyanate group of organosulfur compounds obtained from cruciferous vegetables [106]. It showed promising behavioral cognitive impairments and attenuated brain Aβ burden in AD model mice. In addition, sulforaphane prevented Aβ aggregation and tau phosphorylation via Nrf2 activation.

Allicin (Figure 5b), which is obtained from fresh garlic extract, improved endoplasmic reticulum (ER) stress-related cognitive impairments by increasing double-stranded RNA-dependent protein kinase (PKR)-like ER-resident kinase (PERK)/Nrf2 pathway in the hippocampus of AD rat model treated with tunicamycin, an ER stress stimulator [107].

One of the alternative treatments for AD includes organochalcogens. The preventive effect of [(4-*tert*-butylcyclohexylidene)methyl] (4-methoxystyryl)sulfide (BMMS) (Figure 5c) was demonstrated in an AD mouse model induced by scopolamine treatment. The data obtained in this study showed that BMMS pretreatment was able to prevent OS levels increase and Na^+^/K^+^ ATPase activity reduction in the cerebral cortex, as well as the impairment of short- and long-term memory retrieval induced by scopolamine [108].

A number of pieces of evidence have shown a link in pathological mechanisms between ferroptosis and dysfunctional Nrf2 signaling in AD [34]. In fact, the expression of Nrf2 decreases as we age and Nrf2-regulated proteins that are linked to ferroptosis have been shown to be altered in AD [109,110,111,112] Senescence, a pathology associated with aging and AD, elevates also intracellular iron, and causes resistance to ferroptosis [113].

The pharmacological modulation of the Nrf2 signaling pathway remains one of the more optimal approaches for treating ferroptosis-related pathologies [114,115,116].

One study has investigated the effects of icariin, astragalus, and puerarin on the iron content in the cerebral cortex of APPswe/PS1ΔE9 transgenic mice. These compounds have lightened iron overload by reducing oxidative stress and the inflammatory response [117].

**Figure 6:**
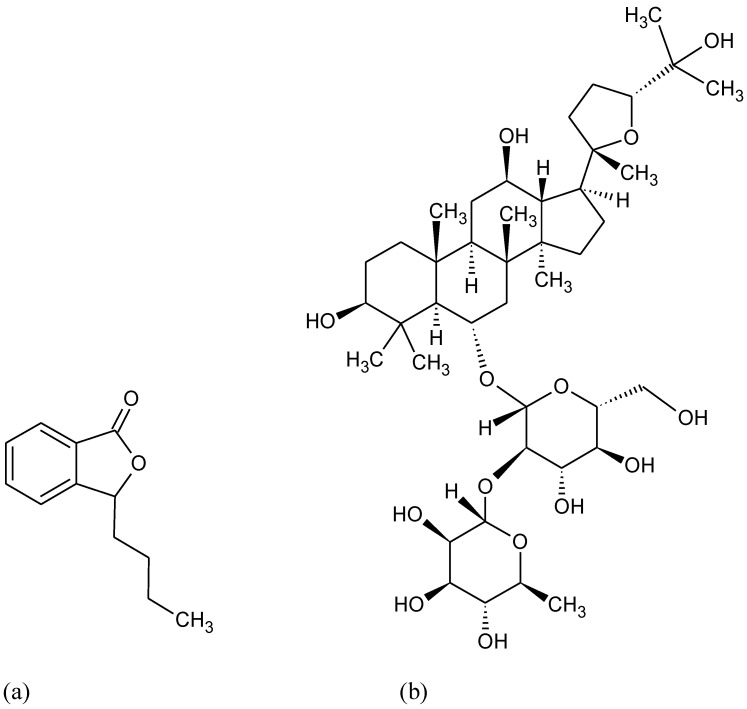
Chemical formula of (**a**) 3-n-butylphthalide and (**b**) pseudoginsenoside-F11.

DL-3-n-butylphthalide (found in celery oil) (Figure 6a), piperine derivative HJ105, Pseudoginsenoside-F11 (found in American ginseng) (Figure 6b) are other compounds that have been demonstrated to be able to recover neuroinflammation and oxidative damage in rat AD models through the Nrf2 signaling pathway activation [118,119,120].

### 4.2. Nrf2 in Parkinson’s Disease

PD is a long-term degenerative disease of the central nervous system that primarily affects the motor system described by rigidity, resting tremor, bradykinesia, and postural instability [121]. These symptoms derive from neurodegeneration of dopaminergic (DAergic) neurons in substantia nigra pars compacta (SNpc), reduction of dopamine levels in the dorsal striatum [122], and intraneuronal accumulations of a-synuclein into Lewy body inclusions [123].

**Figure 7:**
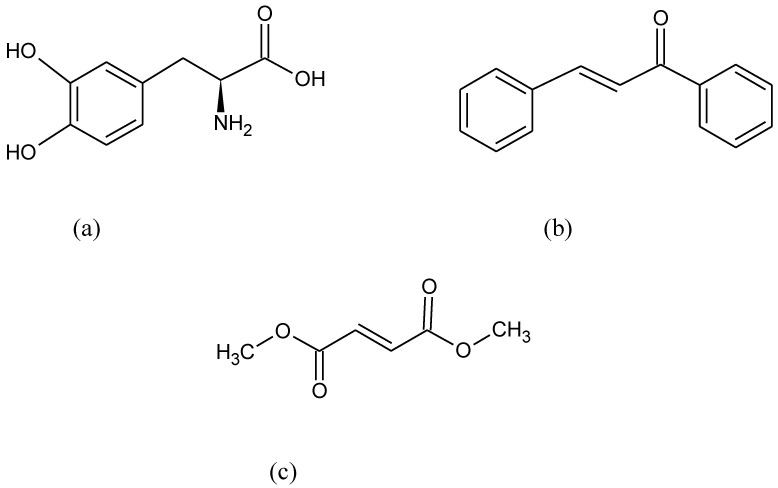
Chemical formula of (**a**) L-dopa, (**b**) chalcone, and (**c**) dimethyl fumarate.

DAergic neurons are particularly affected by OS-related injuries since they can generate large amounts of ROS as a metabolic by-product via metabolization of dopamine by MAO and via auto-oxidation [124]. OS is regularly stated as a hallmark feature of PD [125]. Indeed, several studies have demonstrated increased markers of oxidative damage along with decreased levels of antioxidants in the blood and CSF of PD patients, which was found to be linked with the Nrf2 pathway [126,127]. Nrf2 translocates to the nuclei of DAergic neurons and escalates the transcription of target genes such as HO-1 and NQO1, which are found in the brains of PD patients [128]. The stimulation of Nrf2, which battles OS, appears to be a favorable approach for maintaining cell homeostasis [129]. Levodopa (L-Dopa) (Figure 7a) is the most effective drug for lessening the symptoms of PD. However, long-term usage of L-dopa prompts unwanted effects and can contribute to ROS generation [130].

Chalcones with a common chemical scaffold of 1,3-diaryl-2- propen-1-one, are richly existent in nature with a wide variety of pharmacological activities. Chalcone-type compounds (Figure 7b) stimulate Nrf2 by modifying the cysteine residues of Keap1 by Michael addition since they contain an *α*,*β*-unsaturated carbonyl group. The Nrf2 activator compounds that contain an *α*,*β*-unsaturated carbonyl group display powerful antioxidant and anti-inflammatory effects [131,132,133].

The vinyl sulfones by presenting halogens and nitrogen heterocycle are capable to amplify the activity on Nrf2 and the most effective molecules have halogen substitution in the *ortho* position of the benzene ring, but also a substitution of the vinyl group in *beta*-position with a halogenated pyridine ring [134].

One derivative, (*E*)-3-chloro-2-(2-((2-chlorophenyl)sulfonyl)vinyl)pyridine, significantly exhibited potent Nrf2 activating efficacy, a remarkable increase of Nrf2 nuclear translocation, and Nrf2 protein levels in microglial BV-2 cells. Additionally, this molecule protected DAergic neurons and restored the PD-associated motor dysfunction in the 1-methyl-4-phenyl-1,2,3,6-tetrahydropyridine (MPTP)-induced neurotoxicity mouse model.

Stimulation of Nrf2 was succeeded at the basal ganglia by dimethyl fumarate (Figure 7c) which is known as an MS drug [135]. It was shown that dimethyl fumarate keeps nigral DAergic neurons and reduces astrocytosis and microgliosis. This result points out that targeting Nrf2 with dimethyl fumarate is a promising approach to strengthen the endogenous brain protection process against PD-associated synucleinopathy.

**Figure 8:**
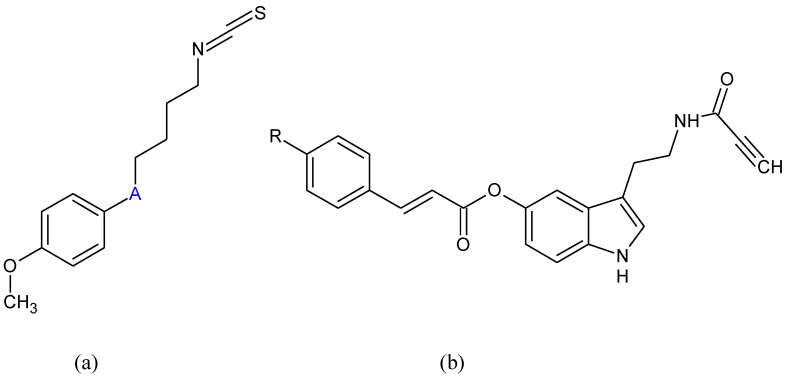
Chemical formula of (**a**) isothiocyanate (A: S=O, C=O, C-OH) derivatives (**b**) indole derivatives (R: H or CH_3_).

Molecules that can be active on both oxidative damage and inflammatory systems have been shown favorable effects against neurodegeneration [136]. Lee [137] showed that isothiocyanate (ITC) (Figure 8a) derivatives induce the expression of antioxidant, Nrf2-dependent enzyme genes avoiding inflammatory reactions in microglia and DAergic neurons in animal models of PD.

2-(1H-indol-3-yl)ethan-1-amine derivatives (Figure 8b) were found as Nrf2 inducers with complementary activities such as selective MAO-B inhibition activity. Some of these molecules showed neuroprotective properties against OS toxicity in PD-related models in vitro [138].

**Figure 9:**
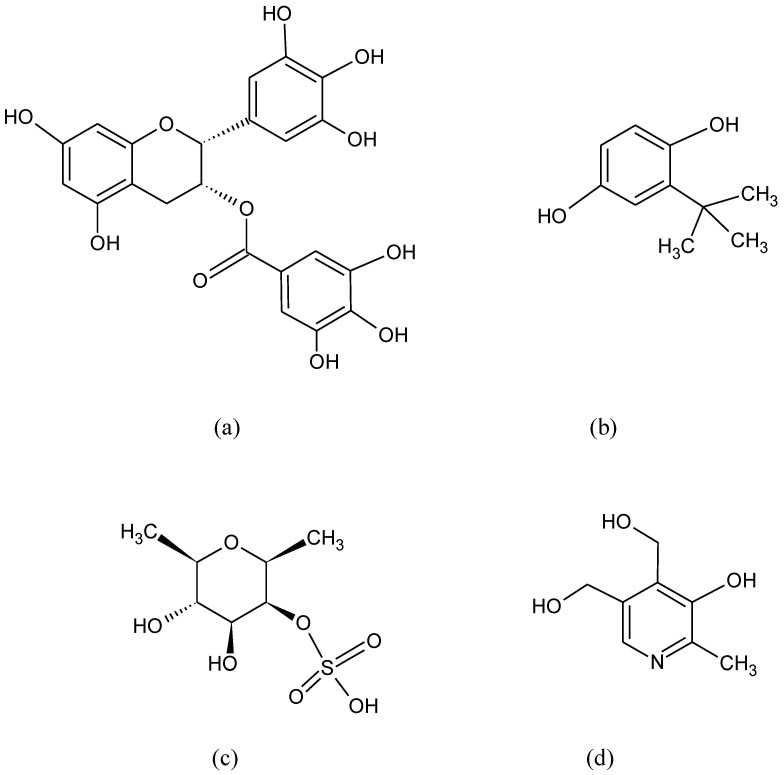
Chemical formula of (**a**) Epigallocatechin gallate, (**b**) Tert-butylhydroquinone (tBHQ), (**c**) Fucoidan, (**d**) Pyridoxine.

Polyphenolic compounds are successful to scavenge free radical species via Nrf2 activation. Epigallocatechin gallate (EGCG) (Figure 9a) is one of the most active catechin compounds. A number of studies have revealed that EGCG is able to cooperate with mitogen-activated protein kinases (MAPK), producing the disassociation of the Nrf2/Keap1 complex [139]. Tert-butylhydroquinone (tBHQ) (Figure 9b) is an oxidized product from butylated hydroxyanisole that becomes electrophilic only when oxidized to tBHQ. In this form, it covalently binds to cysteine residues of Keap1 and stimulates Nrf2 [140]. Fucoidan (Figure 9c), a long chain sulfated polysaccharide present in species of *Brown algae*, significantly improved behavioral deficits and protected DAergic neurons by enhancing the mitochondrial function in a rotenone-induced rat model of PD [141].

In the PD mouse model (MPTP-induced neurotoxicity), the pyridoxine (Figure 9d) treatment has reduced the loss of nigral DAergic neurons facilitating GSH synthesis via the pyruvate kinase M2 (PKM2). This process is triggered by astrocytic dopamine type 2 receptors and mediated by the Nrf2 pathway [142].

The discovery of a missense mutation (A53T) in encoding gene PARK1 in an Italian family, displayed the importance of alpha-synuclein (alpha-SYN) in idiopathic PD [143].

In an animal model lacking the transcription factor Nrf2 (Nrf22/2) the stereotaxic injection of an adeno-associated viral vector for expression of human alpha-SYN in the ventral midbrain induced an increase in nigral dopaminergic neurons degeneration, but also of neuroinflammation and gliosis in comparison to mice expressing Nrf2. The brain tries to compensate for these hallmarks through activation of the Nrf2 pathway. In fact, in the same study, it has been demonstrated an increase in HO-1 expression in astrocytes and microglia in post-mortem brains of patients in early- to middle-stage progression of PD. This study has established the role of Nrf2 in alpha-SYN pathology [144].

Brandes [145] treated neurons isolated from an A53T alpha-synuclein mouse model of synucleinopathy with the methyl ester of fumaric acid (DMF). This treatment reduced ROS levels and improved mitochondrial function and dendritic arborization.

**Figure 10:**
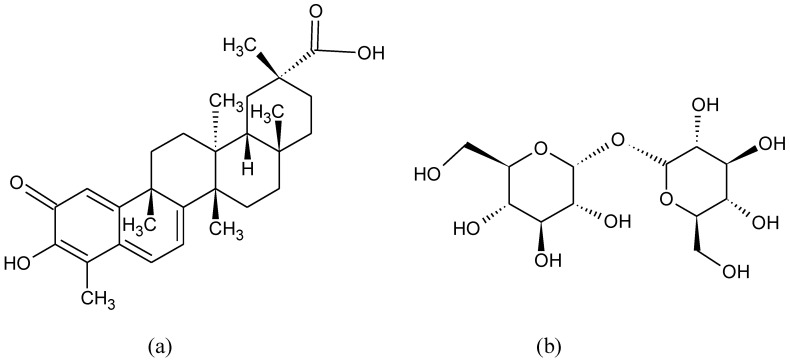
Chemical formula of (**a**) celastrol and (**b**) trehalose.

Celastrol (Figure 10a), a pentacyclic triterpene with anti-inflammatory and anti-oxidative properties, protects through the Nrf2-NLRP3-caspase-1 axis against the neurodegeneration of dopaminergic neurons in an MPTP-induced PD mouse model and relieves Adeno-Associated Virus-mediated human α-SYN overexpression PD model [141].

The alpha-SYN is degraded by both the ubiquitin-proteasome system and the autophagy pathway. As the accumulation of misfolded α-SYN in Lewy bodies is a hallmark of PD, the increased clearance and degradation of this protein represents a different experimental approach toward PD therapy [143].

Trehalose (Figure 10b) is a non-reducing disaccharide found in the hemolymph of invertebrates, but also in bacteria, yeast, fungi, and plants. It protects cells against various environmental stresses including oxidation [146], and it has been shown to enhance autophagy [147] and activate Nrf2 [148,149,150]. Oral trehalose provided in the drinking water has been found to reduce the damage of the substantial nigra dopaminergic neurons, increase autophagy, and up-regulate nuclear translocation of Nrf2 and the expression of downstream antioxidant enzymes in a rat model of PD lesioned with 6-hydroxydopamine [151].

### 4.3. Nrf2 in Multiple Sclerosis

Multiple sclerosis (MS) is a chronic inflammatory neurodegenerative disease characterized by demyelination, astrocytosis, axonal degeneration, and sclerotic plaques as all features of the autoimmune response [152]. Today some therapies have been presented to the clinical practice for the treatment of MS such as the administration of immunosuppressives or treatment with immunomodulatory and neuroprotective drugs [153,154,155]. Unfortunately, the low clinical efficacy of existing molecules justifies the investigation of new pharmacological approaches, including the regulation of redox-sensitive signaling pathways.

**Figure 11:**
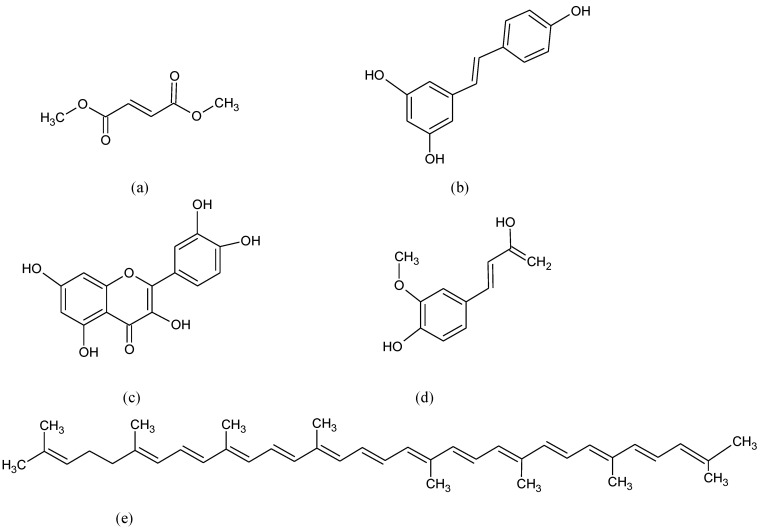
Chemical formula of (**a**) Dimethyl fumarate (DMF), (**b**) resveratrol, (**c**) quercetin, (**d**) ferulic and (**e**) lycopene.

The DMF (Figure 11a) has been approved by the U.S. Food and Drug Administration (FDA) as a treatment of choice for patients with MS (brand name Tecfidera) since 2013. The exact mechanism of action of DMF is not identified yet but it is thought that it can activate the Nrf2 pathway [156,157]. Several Nrf2-activating molecules such as resveratrol, quercetin, ferulic acid, and lycopene (Figure 11b–e) have been shown to reduce LPS-induced neurotoxicity, improve synaptic and mitochondrial function, and inflammatory markers as well as gliosis in MS experimental models [158,159,160].

Ozone therapy also showed an antioxidant and anti-inflammatory activity linked with stimulation of Nrf2 activation in MS patients [161].

### 4.4. Nrf2 in Amyotrophic Lateral Sclerosis

ALS, also known as Charcot’s or Lou Gehrig’s disease, is a severe neurodegenerative condition that is described by advanced upper motor neuron damage in the cerebral cortex and lower motor neuron damage in the brainstem and spinal cord [162]. Research regarding the mechanisms of ALS shows that different dynamics, including excitotoxicity, mitochondrial dysfunction, endoplasmic reticulum stress, neuroinflammation, and OS, can be involved in this process [163]. It is known that OS is activated by an excess production of O_2_^•−^ and NO^•^ in motor neurons and in the central glia, starting from the pre-symptomatic phase of ALS. This may also be assisted by the primary reduction of the GSH level in the several tissues affected by ALS [164].

Immunohistologic evaluation in the lumbar spinal cord of ALS patients revealed augmented 4-hydroxynonenal (HNE)-peroxidated products [165] and malondialdehyde [166]. The 4-HNE, a product from LP of omega-6 polyunsaturated fatty acids, is highly reactive to nucleophilic sites in DNA and proteins inducing cytotoxicity, inactivation of enzymes, and redox imbalance [167]. One of the metabolic pathways involved in the detoxification of 4-HNE is conjugation with GSH catalyzed by GST [168].

In human cervical squamous cancerous cells (HeLa cells), NrfF2 rapidly translocated into the nucleus after exposure to 4-HNE, inducing GST A4 and other enzymes [169]. This process was abolished when Nrf2 was knocked down using small interfering RNA, thus indicating a role of Nrf2 in the detoxification of 4-HNE.

**Figure 12:**
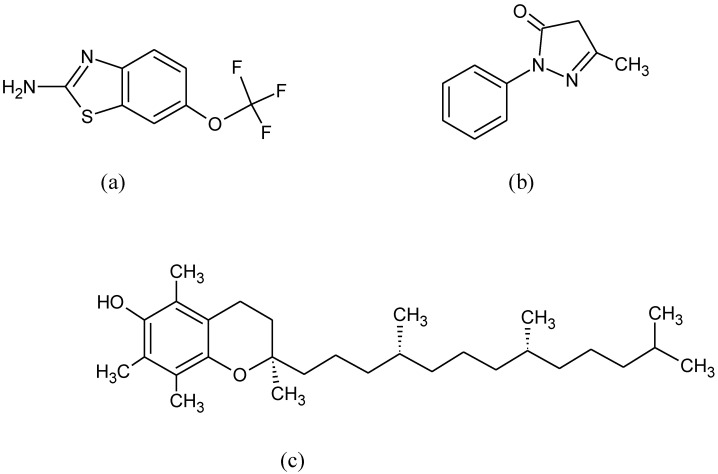
Chemical formula of (**a**) riluzole, (**b**) edaravone and (**c**) vitamin E.

Currently available drugs, riluzole, and edaravone (Figure 12a,b), only extend the survival of ALS patients. Riluzole delays the onset of ventilator-dependence or tracheostomy [170] while edaravone is used to help people to recover from stroke and ALS [171]. Animal studies revealed that edaravone triggers remarkably high expression of Nrf2 and antioxidant defense system in the brain [88]. The role of Nrf2 in ALS is confirmed in ALS mouse models that overexpress Nrf2 exhibiting noteworthy delay at the beginning of ALS and prolonging survival of the animals [172]. Correspondingly, lack of Nrf2 increased immune cell infiltration, glial cell activation in the spine, and inflammatory enzyme in an acute autoimmune model of MS. Furthermore, exacerbated clinical course, a more rapid onset, and a greater percentage of mice with the disease were reported [173].

Vitamin E (Figure 12c) has been shown to be effective in delaying ALS onset in the mouse model, but it was found to be ineffective in some patients with ALS [174]. It remains likely that vitamin E supplements in healthy people may reduce or delay the risk of ALS [175].

**Figure 13:**
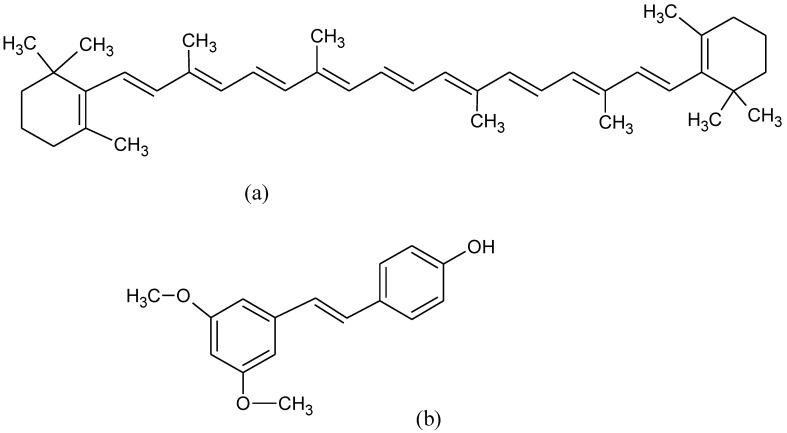
Chemical formula of (**a**) beta carotene and (**b**) pterostilbene.

There is a favorable link between ALS and the intake of carotenes [176]. β-carotene (Figure 13a) may work for treating neuroinflammation and apoptosis in ALS patients [177]. Therefore, carotene intake might be beneficial for the prevention and delaying of the onset of ALS [178]. Epigallocatechin-3-gallate (EGCG), the major bioactive compound of green tea, shows anti-neurodegenerative and antioxidant effects, particularly on the motor neurons since it can cross the blood-brain barrier and modifies mitochondrial responses to OS [179]. Some natural molecules such as flavonoids, resveratrol, and curcumin are beneficial for the treatment of ALS. Their action is associated with the reduction of cognitive damage and neuronal dysfunction and suppresses neuroinflammation [180].

Pterostilbene (PTER) (Figure 13b) (trans-3,5-dimethoxy-4hydroxystilbene), belongs to the stilbenes family as resveratrol but with a better pharmacokinetics profile (excellent oral bioavailability, lipophilicity and higher permeability to targeted tissues) [181]. PTER reduces CNS injury in vitro triggering Nrf2 signaling and exhibiting defensive activity against mitochondrial dysfunction-derived OS [182].

Moreover, PTER protected murine hippocampal neuronal HT22 cells from glutamate-induced OS injury-inducing Nrf2-GSH-SOD pathway [183].

Familial ALS in some cases is due to a mutation of SOD1 and improvement of the NAD+ reverses the toxicity of primary astrocytes expressing the SOD1 mutation related to ALS [184]. In a randomized, double-blind, placebo-controlled study in humans, administration of a combination of nicotinamide riboside, a nicotinamide adenine dinucleotide (NAD+) precursor vitamin, and pterostilbene (PT) increased NAD+ levels [185]. Due to this, PTER could be a promising therapeutic strategy in ALS.

**Figure 14:**
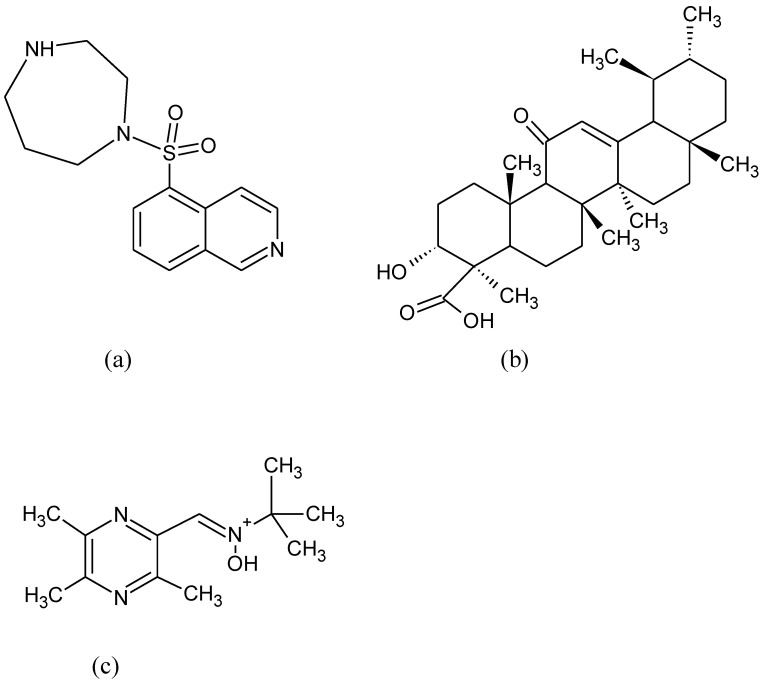
Chemical formula of (**a**) fasudil, (**b**) 11-keto-beta-boswellic acid, and (**c**) tetramethylpyrazine nitrone.

Multitarget hybrid fasudil (Figure 14a) derivatives were found as promising molecules for the treatment of ALS by activating the Nrf2 and stimulating the expression of the antioxidant response enzymes HO-1 and NQO1via a KEAP1-dependent mechanism [186]. Acetyl-11-keto-beta-boswellic acid (AKBA) (Figure 14b) is a pentacyclic triterpenoid mixture obtained from *Boswellia serrata* and other plants, with anti-inflammatory and antioxidant properties [187]. In a rat model of mercury-induced ALS, AKBA treatment restored behavioral, neurochemical, and morphological alterations [188]. Tetramethylpyrazine nitrone (TBN) (Figure 14c) is a derivative of tetramethylapyrazine, that reduced motor deficits and cognitive impairment in the early stages of ALS progression and prolonged survival rate in mice [189].

## 5. Concluding Remarks

In spite of growing studies about the etiology and pathogenesis of neurodegenerative diseases and research on finding effective drugs, the question related to the origin of these diseases remains open, but oxidative damage has gained importance in etiology fields.

Exposure to OS initiates cell injury that causes neurodegeneration due to a lack of the regulation of the inflammatory defense. During the chronic state of OS, ROS/RNS induce the constant stimulation of the signaling pathways. Since it was revealed that Nrf2 is a major regulator of oxidant resistance, it has been associated with a range of chronic diseases such as neurodegenerative disorders that are characteristically related to OS.

By modifying the oxidant system, Nrf2 contributes to the managing of several vital functions, such as ferroptosis, inflammasome, and autophagy opening new approaches for drug discovery

Since OS, together with neuroinflammation and mitochondrial dysfunction, is the most important hallmark of neurodegenerative conditions, a molecular involvement in Nrf2/ARE signaling and the development of the transcriptional action of specific genes are targets for avoidance or suspending the beginning of age-associated and genetic neurogenerative disorders [190].

Studies have been dedicated to recognizing the processes that control the relationship between Nrf2 and Keap1 and there are some molecules described as Nrf2 activators in neuroinflammation [106]. Dimethyl fumarate (DMF) is the only drug approved by FDA as an effective molecule in MS [191]. Nrf2 is the main modulator for the two essential cytoprotective pathways, anti-inflammation and antioxidation. Even though numerous studies are now pointing to Keap1—the main controller of Nrf2—it is still not clear enough to develop these agents pointedly against neurodegenerative conditions. Recent data propose that antioxidant molecules power for triggering Nrf2/ARE pathways and autophagy signaling, revealed to increase the expression of Nrf2/ARE, evidence protecting in many studies [192].

At present, the trouble related to the management of neurodegenerative disorders is their multifaceted pathogenesis. Monitoring only one target is not sufficient for efficacious treatment. The definition of an association between activity and physicochemical property of Nrf2/ARE is significantly important. Ensuring satisfactory BBB penetration, reducing the activity of the Keap1-Nrf2. Due to the extensive dissemination of Nrf2 in vivo, Nrf2 activators with a high BBB penetration and CNS directing capability are beneficial for the cure of neurodegeneration [193]. Comprehensive studies are essential for rationally activating Nrf2 to deliver, active, harmless, and manageable approaches for neurodegenerative disorders.

It is clear that ROS work as serious intracellular signaling agents but overproduction can lead to OS and impairment of vital macromolecules, ultimately causing cell death. Nrf2 is significant protection, especially for neurons. Nrf2 expression and action are lessened in humans and animals which were observed from numerous experimental data. Triggering the activation of Nrf2 displays favorable outcomes in many animal models of neurodegenerative conditions. Stimulation of Nrf2 has been connected with neuroprotection. A better understanding of the regulatory processes of Nrf2 action will help to discover novel compounds to avoid, slow down, or perhaps cure many neurodegenerative diseases.

## Data Availability

Data sharing not applicable.
